# Interrogation of the perturbed gut microbiota in gouty arthritis patients through in silico metabolic modeling

**DOI:** 10.1002/elsc.202100003

**Published:** 2021-06-09

**Authors:** Michael A. Henson

**Affiliations:** ^1^ Department of Chemical Engineering and the Institute for Applied Life Sciences University of Massachusetts Amherst MA USA

**Keywords:** bacterial communities, gout, gut microbiota, machine learning, metabolic modeling

## Abstract

Recent studies have shown perturbed gut microbiota associated with gouty arthritis, a metabolic disease characterized by an imbalance between uric acid production and excretion. To mechanistically investigate altered microbiota metabolism associated with gout disease, 16S rRNA gene amplicon sequence data from stool samples of gout patients and healthy controls were computationally analyzed through bacterial community metabolic models. Patient‐specific community models constructed with the metagenomics modeling pipeline, mgPipe, were used to perform k‐means clustering of samples according to their metabolic capabilities. The clustering analysis generated statistically significant partitioning of samples into a *Bacteroides*‐dominated, high gout cluster and a *Faecalibacterium*‐elevated, low gout cluster. The high gout cluster was predicted to allow elevated synthesis of the amino acids D‐alanine and L‐alanine and byproducts of branched‐chain amino acid catabolism, while the low gout cluster allowed higher production of butyrate, the sulfur‐containing amino acids L‐cysteine and L‐methionine, and the L‐cysteine catabolic product H_2_S. By expanding the capabilities of mgPipe to provide taxa‐level resolution of metabolite exchange rates, acetate, D‐lactate and succinate exchanged from *Bacteroides* to *Faecalibacterium* were predicted to enhance butyrate production in the low gout cluster. Model predictions suggested that sulfur‐containing amino acid metabolism generally and H_2_S more specifically could be novel gout disease markers.

**Abbreviations**
COBRA: constraint‐based reconstruction and analysis
EUD: average EU diet
FDR: false discovery rate
FVA: flux variability analysis
HFD: high fiber diet
HPD: high protein diet
NMPC: net maximal production capability
OTU: operational taxonomic unit
VMH: virtual metabolic human

## 1 INTRODUCTION

The human gut microbiota play essential roles in digestion of plant polysaccharides [1, 2], synthesis of essential and health‐promoting metabolites [3, 4], development of host immune response [5], and maintenance of colonization resistance to pathogens [6]. Numerous disease processes have been correlated with disruptions of the gut microbiota composition, often termed dysbiosis [10, 11, 12]. Microbiota‐associated diseases range from direct ailments of the gut such as inflammatory bowel disease [13] and *Clostridioides difficile* infection [14], to general metabolic diseases such as diabetes [15] and obesity [16], to systemic aliments such as cardiovascular disease [17], and even to neurological disorders such as depression [18] and Parkinson's disease [19]. The relative abundances of the diverse bacterial taxa that comprise the gut microbiota can be determined from stool samples through the application of 16S rRNA gene amplicon sequencing [7, 8, 9]. While studies that correlate changes in microbiota abundances to disease development have revolutionized our understanding of human disease, such compositional‐based analyses often provide little information about the underlying mechanisms by which the microbiota may drive and/or respond to disease processes.

Gouty arthritis is a metabolic disease related to the inability of the human host to properly regulate uric acid, a primary metabolite of purine metabolism [20, 21, 22]. As the uric acid concentration in the blood serum exceeds ∼400 μmol/L (termed hyperuricemia) [23, 24], susceptible individuals may begin to suffer gout symptoms including painful inflammation due to the deposition of uric acid crystals in joints [25, 26]. Therapeutic treatments include drugs such as Allopurinol and Febuxostat that reduce host uric acid synthesis, Krystexxa that increases the breakdown of uric acid to urea, Probenecid and Lesinurad which increase uric acid excretion, and a broad array of anti‐inflammatory compounds [27, 28]. Several recent studies in humans [29, 30, 31] and murine models [32, 33, 34, 35, 52] have correlated changes in gut microbiota composition to the presence of gout disease, suggesting that microbiota properties could be used to monitor disease development, progression and recovery. Several of these 16S‐based studies have been combined with gene catalog [36] and metabolomic [29] analyses to better understand metabolic changes that accompanied compositional dysbiosis. While they provided new insights into the association between gout disease and altered gut microbiota, these studies were inherently limited in their ability to quantify the functional metabolic differences between the gut communities of gout patients versus healthy controls.

**PRACTICAL APPLICATION**

Uric acid is produced in the human body from purine compounds contained in dietary meat, poultry and seafood. Gouty arthritic is a chronic metabolic disease in which elevated levels of uric acid in the blood result in crystal formation, deposition in joints and chronic inflammation. Recent experimental studies have shown that the gut microbiota are perturbed in gout disease, suggesting that altered microbiota metabolism may result from gout development. Building on these experimental results, this study used computational metabolic modeling to investigate altered microbiota metabolism associated with gout disease. Patient‐specific models of gut bacterial communities were constructed and analyzed to predict altered production of metabolites derived from the microbiota in gout patient versus healthy controls. The methodology identified butyrate, a metabolite known to promote gut health, sulfur‐containing amino acids and hydrogen sulfide, a metabolite known to promote inflammation, as possible metabolic markers of gout disease.

This in silico computational study was based on the hypothesis that altered gut microbiota were the result rather than the cause of gout disease, as a causative role has not been demonstrated to date. Indeed, uric acid in mainly produced in the liver by nucleic acid catabolism and only about 20% of uric acid production occurs from digestion of purine‐rich foods [35]. Furthermore, only about 30% of host generated uric acid is excreted through the intestine, with the remainder excreted from the kidneys [35]. Although altered uric acid metabolism in the gut microbiota of gout patients has been suggested [36], such perturbations are unlikely to be the major cause of elevated uric acid levels in the blood. Consequently, this study focused on the possibility of using predicted gut microbiota properties as clinically‐relevant signatures of gout disease rather than as treatable disease drivers.

Consistent with this hypothesis, published 16S abundance data derived from patient stool samples [36] were used to build sample‐specific computational models for identifying microbiota‐synthesized metabolites that may be produced at differing levels in gout patients compared to healthy controls. The 16S dataset, which included bacterial taxa abundances for 41 gout patients and 42 healthy controls, was processed using a metagenomics modeling pipeline (mgPipe; [37]) to construct community metabolic models that spanned 50 taxa (48 genera and two families). The computational models were simulated using three in silico diets, and the resulting simulation data was subjected to machine learning and statistical analyses to correlate metabolic function and patient type, extract information about metabolite synthesis capabilities at the community and individual taxa levels, predict intra‐taxa metabolite crossfeeding relationships and explore the impact of dietary nutrient levels on community metabolism.

## 2 MATERIALS AND METHODS

### 2.1 Patient data

Gut microbiota composition data were obtained from a published study [36] in which stool samples from 83 patients were subjected to 16S rRNA gene amplicon library sequencing. The study included 41 gout patients as determined by clinical symptoms and elevated blood uric acid levels and 42 healthy controls (Table 1). The patients ranged in age from 27 to 75 years (average 49.1 years) and contained 41% females. For each patient sample, 16S‐derived relative bacterial abundances were provided at different taxonomic levels that included 15 phyla, 28 classes, 38 orders, 71 families, and 129 genera. Extensive clinical metadata, including the blood uric acid concentration, also were provided for each sample (Table S1).

**TABLE 1:** Summary of patient metadata from [36]

	Gouty	Healthy	Total
Patients	41	42	83
Age (years)	49.4	48.7	49.1
Female/Male	17/24	17/25	34/49
Body Mass Index (kg/m^2^)	23.1	23.2	23.2
Blood Uric Acid (μmol/L)	496	242	368
Urea Nitrogen (mmol/L)	7.78	4.48	6.11
Blood Glucose (mmol/L)	5.76	5.17	5.46

### 2.2 Community metabolic modeling

Community metabolic models were restricted to 50 taxonomic groups to limit the computational effort associated with model building, simulation and analysis while achieving adequate coverage of the 16S operational taxonomic unit (OTU) read data. The models accounted for the 48 most abundant genera across the 83 samples subject to the requirement that each genus could be modeled using genome‐scale metabolic reconstructions available in the Virtual Metabolic Human (VMH) database ([61]; www.vmh.life). Combined reads for *Escherichia*/*Shigella* were equally split between the two genera. Because unidentified *Lachnospiraceae* and *Ruminococcaceae* accounted for 4.9% and 1.1% of total reads, respectively, these two families were included in the models and combined with unmodelable genera (*Lachnobacterium*, *Anaerosporobacte*r, *Parasporobacterium*, *Hespellia* and *Robinsoniella* for *Lachnospiraceae*; *Oscillibacter*, *Anaerofilum*, *Acetivibrio*, *Acetanaerobacterium*, *Sporobacter*, and *Hydrogenoanaerobacterium* for *Ruminococcaceae*). This procedure resulted in 50 modeled taxa that accounted for an average of 97.0% of total OTU reads across the 83 samples (Table S1).

For each sample, the reads associated with each taxon were normalized to unity by dividing by the sum of reads. These normalized taxa abundances were used to construct sample‐specific community metabolic models. The function create Pan Models within the metagenomics pipeline (mgPipe; [37]) of the MATLAB Constraint‐Based Reconstruction and Analysis (COBRA) Toolbox [62] was used to construct genus‐ and family‐level models from the 818 strain models available in the VMH database. According to function documentation available on the COBRA website, the function create Pan Models combined all reactions for strains belonging to the taxon of interest and attempted to remove futile cycles that may result from the combined reactions by making certain reactions irreversible. Because the 16S data did not provide resolution at the species and strain levels, the community metabolic models were unable to account for differences in community composition and function below the genus level. Despite this limitation, the simulation results showed that the pan‐genome metabolic models used for community modeling allowed substantial differentiation of samples according to their functional capabilities.

The function initMgPipe was used to construct a community metabolic model for each of the 83 patient samples. Model construction required specification of normalized taxa abundances for each sample and maximum uptake rates of dietary nutrients, which were specified according to EU average, high protein and high fiber diets downloaded from the VMH databse (Table S2). The community models contained an average of 38,570 reactions (minimum 22,099; maximum 57,820). All models contained the same constraints for the maximum nutrient uptake rates specified for the chosen diet, while each model had different constraints imposed for the sample taxa abundances.

Following the model building process, mgPipe automatically performed flux variability analysis (FVA) for each model with respect to each of the 409 metabolites assumed to be exchanged between the microbiota and the lumen and fecal compartments. FVA calculations were performed with the COBRA code fastFVA using the CPLEX linear program solver to maximize/minimize the production of the metabolite and to maximize/minimize the uptake of the metabolite subject to the additional constraint that the community biomass flux remained in the range 0.4–1.0 mmol/day [63]. The FVA results were used within mgPipe to compute the net maximal production capability (NMPC; [63]) of each metabolite by each model. Each NMPC value was calculated as the absolute value of the sum of two computed FVA solutions, the first which maximized metabolite secretion into the fecal compartment and the second which maximized metabolite uptake from the lumen compartment. Each NMPC value represented the sample‐specific potential for community production of a single metabolite given the applied nutrient uptake and biomass flux constraints. The mgPipe framework is based on analysis of metabolite‐specific NMPCs across samples to assess the capabilities of modeled communities to differentially produce metabolites. Due to the nature of FVA calculations, the reported NMPCs do not imply that maximal production of multiple metabolites may be achieved simultaneously. In this study, each NMPC was calculated and analyzed independently for each modeled sample. Furthermore, NMPCs were calculated for three different diets to assess the possible impact of nutrient levels on community metabolism (Tables S3‐S5).

Unfortunately, mgPipe currently does not offer the capability to directly extract the metabolite synthesis capabilities of each modeled taxa within a sample. This information was deemed important to understand which taxa were contributing to metabolite synthesis and the intra‐taxa crossfeeding relationships which supported maximal production of a particular metabolite. Therefore, specialized MATLAB scripts were written to utilize available mgPipe functions (e.g., adaptVMHDietToAGORA, guidedSim, useDiet) to perform FVA with respect to selected metabolites and to extract taxa‐level secretion and uptake fluxes. The complete metabolic modeling workflow can be viewed as complementary to more established metagenomic analysis techniques such as Phylogenetic Investigation of Communities by Reconstruction of Unobserved States (PICRUSt; [64]) by quantifying community interactions such as nutrient competition, metabolite crossfeeding and product synthesis.

### 2.3 Data analysis

Patient data consisted of normalized taxa abundances and model‐predicted data consisted of diet‐dependent NMPCs, both of which could be connected to associated metadata on a sample‐by‐sample basis (Table S1). Data analysis were limited to samples in which the modeled taxa accounted for at least 90% of the unnormalized abundances (39/41 gouty samples, 39/42 healthy samples) to achieve adequate representation of the original 16S gene amplicon data. Both normalized taxa abundances and model‐predicted NMPCs were subjected to unsupervised machine learning techniques including clustering and principal component analysis (PCA) to extract putative relationships between partitioned samples and patient gout status. Rather than apply supervised learning to samples partitioned based on their known clinical status (i.e., gouty, healthy), unsupervised learning was performed to determine if samples clustered by taxa abundances or NMPCs could be associated with gout status. This approach was applied under the hypothesis that clustering could partially unravel the complex gout disease etiology and reveal at least one cluster with statistically high levels of gouty or healthy samples.

Clustering was performed using the MATLAB function k means with the squared Euclidean distance metric, the k‐means++ algorithm for cluster center initialization [65] and 1000 replicates. When clustering was applied to NMPC data generated from the average EU diet, an optimal number of three clusters was determined using the MATLAB function evalclusters with the k means clustering method, the silhouette evaluation criterion [66], the sum of absolute difference as the distance measure and 100 replicates. To facilitate subsequent comparisons, three clusters also were used for the high protein and high fiber diets and for clustering of abundance data. For each in silico diet tested, the clustering method proved robust in that the same clustered samples were consistently returned despite the randomness of cluster initialization and the existence of local minima [67].

PCA was performed directly on normalized taxa abundances and model‐predicted NMPC data rather than on data preprocessed with sample dissimilarity measures such as the Bray–Curtis [68] or UniFrac [69] metrics. This approach was deemed appropriate since PCA was used for preliminary data visualization and not quantitative data analysis. Statistical significance of associations between categorial variables (e.g., gouty/healthy) across sample groups were assessed using Fisher's exact test [70]. Correlations between taxa based on their abundances across samples were calculated using the proportionality coefficient [71], which accounts for the effects of data normalization. Statistically significant differences between NMPCs across samples were assessed using the Wilcoxon rank‐sum test [72]. The resulting *p*‐values were used to calculate the false discovery rate (FDR) for each metabolite using the MATLAB function mafdr with the Benjamini‐Hochberg method [73].

## 3 RESULTS

### 3.1 Samples clustered by metabolic capability were associated with patient type

Prior to metabolic modeling, the 16S‐derived abundance data were analyzed directly to identify community compositional features associated with gouty and healthy sample. Data analysis were limited to samples in which the modeled taxa accounted for at least 90% of the unnormalized abundances (39/41 gouty samples, 39/42 healthy samples; see Materials and Methods). Among the 25 most abundant taxa across the 78 samples, the abundances of six taxa were significantly different (Wilcoxon rank‐sum test, FDR <0.05) between the 39 gouty and 39 healthy samples. Notably, *Faecalibacterium* was significantly elevated (FDR = 3 × 10^−4^) in the healthy samples (average abundance 0.144) compared to the gouty samples (average abundance 0.063; Figure S1A). Five taxa including three butyrate producers (*Faecalibacterium*, *Coprococcus*, *Roseburia*) were most negatively correlated with the blood uric acid concentration across the 78 samples, while three other taxa (*Parabacteroides*, *Clostridium*, *Veillonella*) were most positively correlated with blood uric acid (Figure S1B). *Faecalibacterium* was most positively correlated to three butyrate producers (*Coprococcus*, *Roseburia*, *Subdoligranulum*) and *Akkermansia* as measured by the proportionality coefficient (Figure S1C). A principal component plot of the taxa abundances showed no clear delineation of gouty versus healthy samples (Figure S1D). Taken together, these results support the conclusion in the original experimental study [36] that depletion of *Faecalibacterium* and other butyrate producers was associated with gout development.

**FIGURE 1:**
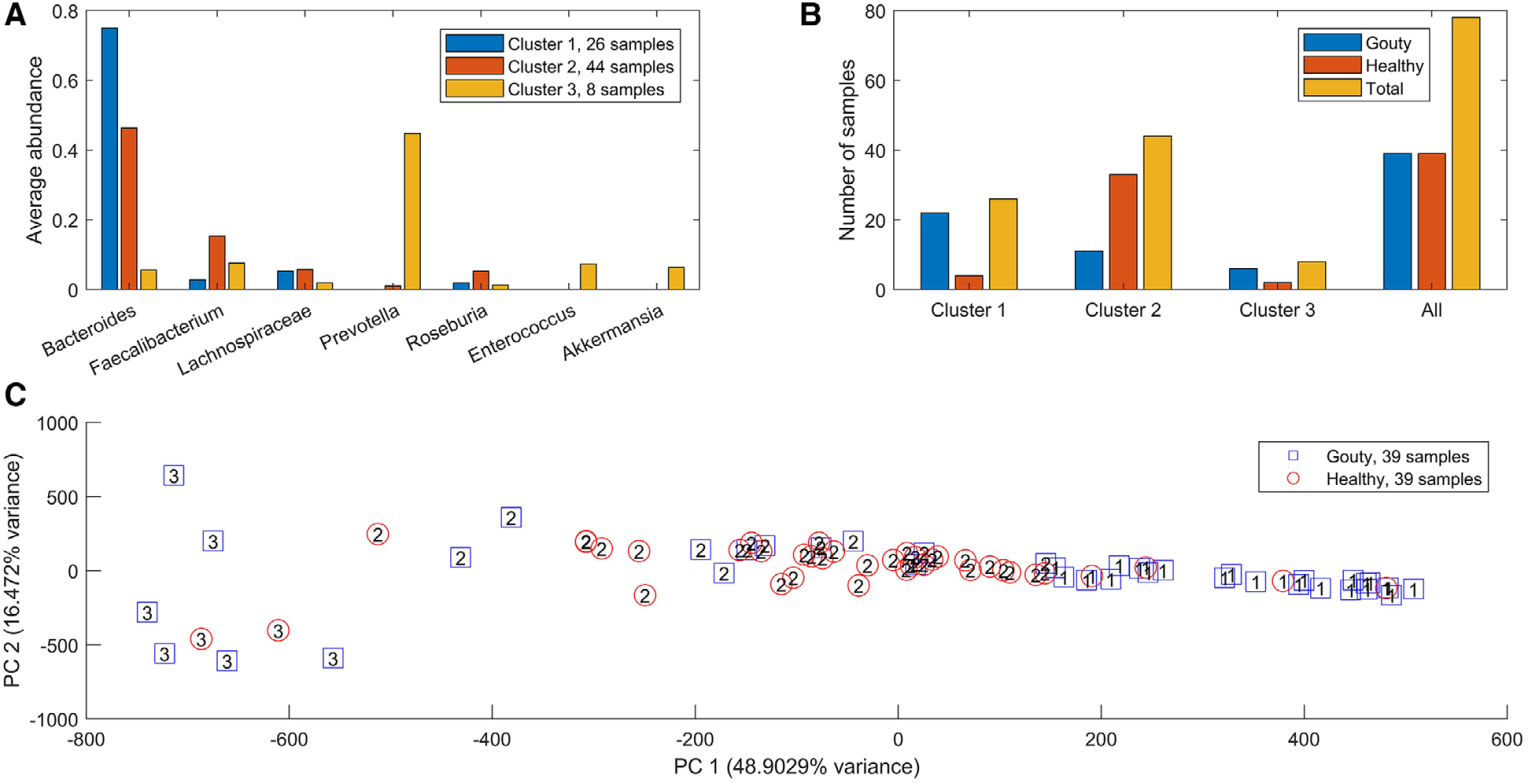
Clustering of model‐predicted NMPCs obtained with an average EU diet. (A) Average abundances of taxa which averaged at least 5% across at least one cluster. (B) Number of gouty, healthy and total samples in each cluster. Cluster 1 contained a disproportionate large number of gouty samples compared to cluster 2 (*p* < 10–5) and the entire dataset (*p* = 0.002). Cluster 2 contained a disproportionate large number of healthy samples compared to cluster 3 (*p* = 0.01) and the entire dataset (*p* = 0.008). (C) Principal component plot of the model‐predicted NMPCs with gouty and healthy patient samples labeled by their associated clusters

Using an in silico European diet (Table S2), the net maximal production rates (NMPCs) predicted for 409 exchanged metabolites across the 78 samples were clustered to investigate if model‐predicted metabolic capabilities were associated with sample type. Three clusters produced a group of 26 samples dominated by *Bacteroides* (average abundance 0.75; Figure 1A), a group of 44 samples with elevated *Faecalibacterium* (average abundance 0.15), and a small group of eight samples with elevated *Prevotella* (average abundance 0.45). The *Bacteroides*‐dominated cluster contained a disproportionately large number of gouty samples (22/26) compared to the *Faecalibacterium*‐elevated cluster (11/44, *p* < 10^−5^; Figure 1B) and to the entire sample set (39/78, *p* = 0.002). Similarly, the *Faecalibacterium*‐elevated cluster contained a disproportionately large number of healthy samples (33/44) compared to *Prevotella*‐elevated cluster (2/8, *p* = 0.011) and the entire sample set (39/78, *p* = 0.008). A principal component plot of the model‐predicted NMPCs showed the *Prevotella*‐elevated cluster samples as outliers and clearly identified the *Bacteroides*‐dominated cluster samples as disproportionally gouty. Interestingly, gout patients have been reported to have elevated abundances of *Prevotella intermedia* in the oral microbiota [38]. Taken together, these results suggested that elevated *Bacteroides* abundance may result from the gout disease process.

### 3.2 Metabolic modeling predicted differential synthesis of amino acids and fermentation products

Due to the small number of samples contained in the *Prevotella*‐elevated cluster (also called the medium gout cluster with 6/8 = 0.75 fraction of gouty samples), further statistical analyses were focused on the *Bacteroides*‐dominated cluster (also called the high gout cluster with 22/26 = 0.85 fraction of gouty samples) and the *Faecalibacterium*‐elevated cluster (also called the low gout cluster with 11/44 = 0.25 fraction of gouty samples). A rank sum test (FDR <0.05), which assessed differences in median values, was performed to identify metabolites with the potential to be differentially produced in the high and low gout clusters. To reduce the 106 metabolites identified to a more manageable number, each metabolite also was required to have an average production rate of >10 mmol/d in at least one cluster and to exhibit at least 10% difference between the mean production rates in the two clusters. The resulting set of 42 differentially produced metabolites covered a wide range of metabolic pathways and included the amino acids D‐alanine, L‐alanine, L‐cysteine, L‐histidine, L‐isoleucine, L‐methionine and L‐tyrosine as well as common products of gut microbiota fermentation such as butyrate, H_2_, H_2_S, isobutyrate, isocaproate, isovalerate and L‐lactate (Figure S2, Table S6). Interestingly, hypoxanthine was the only metabolite directly involved in purine metabolism that was predicted to be differentially produced between the two clusters, supporting the hypothesis that gut microbiota were not the main drivers of gout disease. Similar predictions were obtained when the samples were partitioned directly according to their clinical status (Table S6), suggesting that sample clustering according to metabolite production capabilities captured the dominant metabolic features differentiating gouty and healthy samples.

**FIGURE 2:**
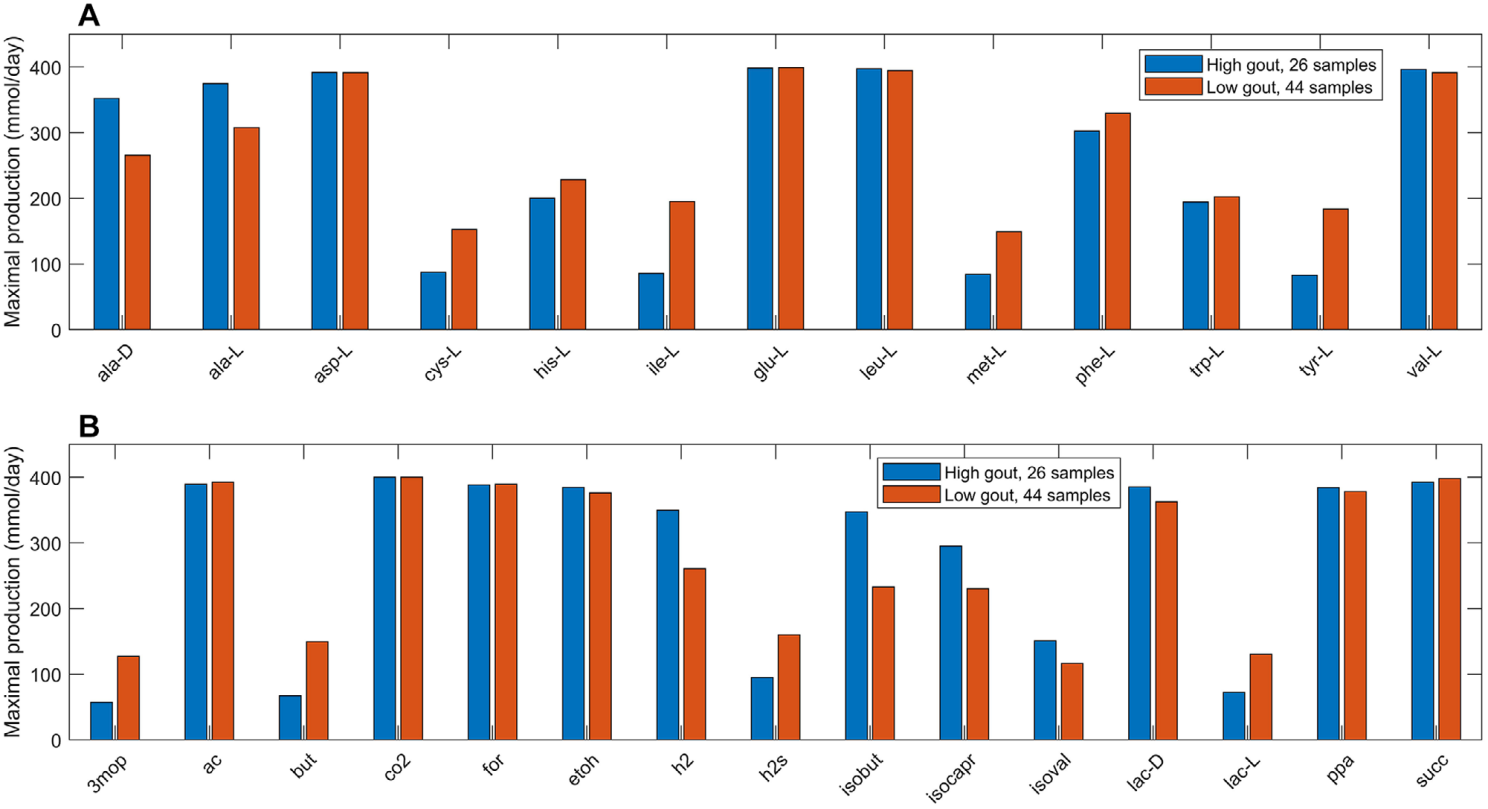
Maximal amino acid and fermentation byproduct synthesis capabilities in the high and low gout clusters from an average EU diet. (A) Classes of amino acids sharing common metabolic pathways and containing at least one amino acid differentially produced between the high and low gout clusters. (B) Common metabolic byproducts of carbohydrate fermentation and amino acid catabolism. Metabolite abbreviations are taken from the VMH database (www.vmh.life)

Further computational analyses were performed on the seven differentially produced amino acids along with six additional amino acids that shared metabolic pathways with these seven amino acids and the eight differentially expressed fermentation products along with seven additional byproducts commonly produced by gut microbiota. The high gout cluster was predicted to have significantly elevated capabilities for production of alanine, H_2_ and three products of branched‐chain amino acid catabolism (isobutyrate from valine, isocaproate, and isovalerate from leucine; Figure 2). By contrast, the low gout cluster was characterized by the potential for significantly elevated production of butyrate, L‐lactate, the sulfur‐containing amino acids L‐cysteine and L‐methionine, the L‐cysteine catabolic product H_2_S, L‐isoleucine and its catabolic product 3‐methyl‐2‐oxovaleric acid, L‐histidine, and L‐tyrosine. Model predictions of elevated alanine and reduced butyrate metabolism in gout patients compared to healthy controls were consistent with gene catalog and metabolomic studies [29, 31, 36]

**FIGURE 3:**
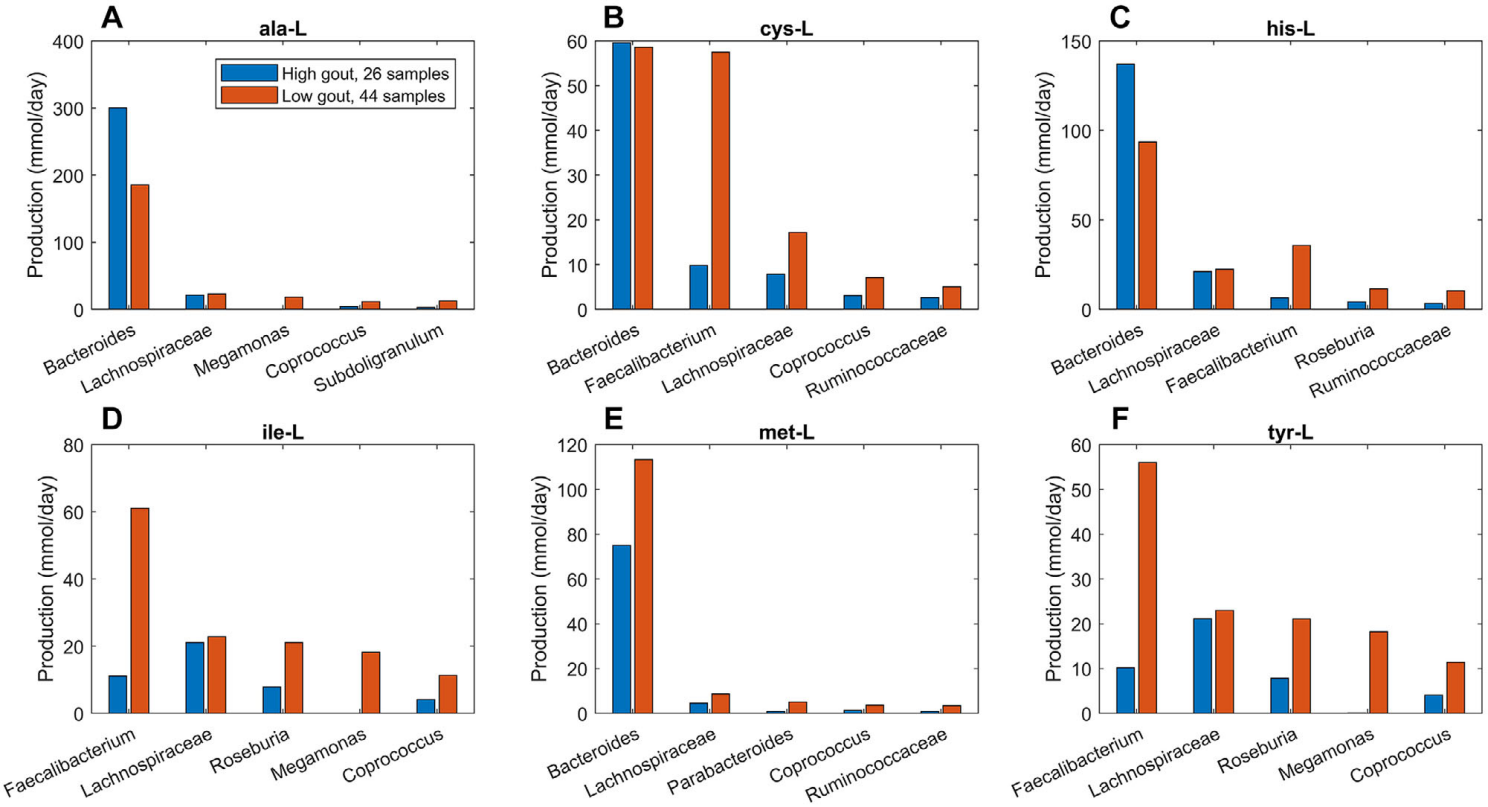
Individual taxa contributions to maximal synthesis of amino acids differentially produced between the high and low gout clusters from an average EU diet. The amino acids shown from top left to bottom right are L‐alanine, L‐cysteine, L‐histidine, L‐isoleucine, L‐methionine, and L‐tyrosine. D‐alanine has been omitted for brevity. For each amino acid, the top five taxa are shown in the order of their total production across the two clusters

To further investigate metabolite production capabilities of the gout‐ and health‐associated gut communities, the contributions of individual taxa to maximal synthesis of differentially produced amino acids and fermentation byproducts were computed. *Bacteroides* was responsible for enhanced D‐alanine, L‐alanine and L‐histidine production in the high gout cluster samples (Figure 3), which were characterized by high *Bacteroides* abundances. The production of L‐isoleucine and L‐tyrosine, two amino acids not secreted by the *Bacteroides* metabolic model, were elevated in the low gout cluster due to increased synthesis by more abundant butyrate‐producing taxa (*Faecalibacterium*, *Lachnospiraceae*, *Roseburia*, *Coprococcus*) as well as by *Megamonas*. Interestingly, *Bacteroides* was predicted to have similar L‐cysteine and reduced L‐methionine synthesis capabilities in the high gout cluster despite these samples having relatively high *Bacteroides* abundances. This behavior resulted in significantly reduced total production of L‐methionine and L‐cysteine, which were also synthesized by *Faecalibacterium* and other butyrate producers, in the high gout cluster. These predictions suggested a possible role for sulfur‐containing amino acids in the perturbed microbiota of gout patients. Synthesis of the six non‐differentially produced amino acids was similar between the two clusters because elevated synthesis by *Bacteroides* in the high gout cluster was balanced with increased synthesis by butyrate producers and *Megamonas* in the low gout cluster (Figure S3).

**FIGURE 4:**
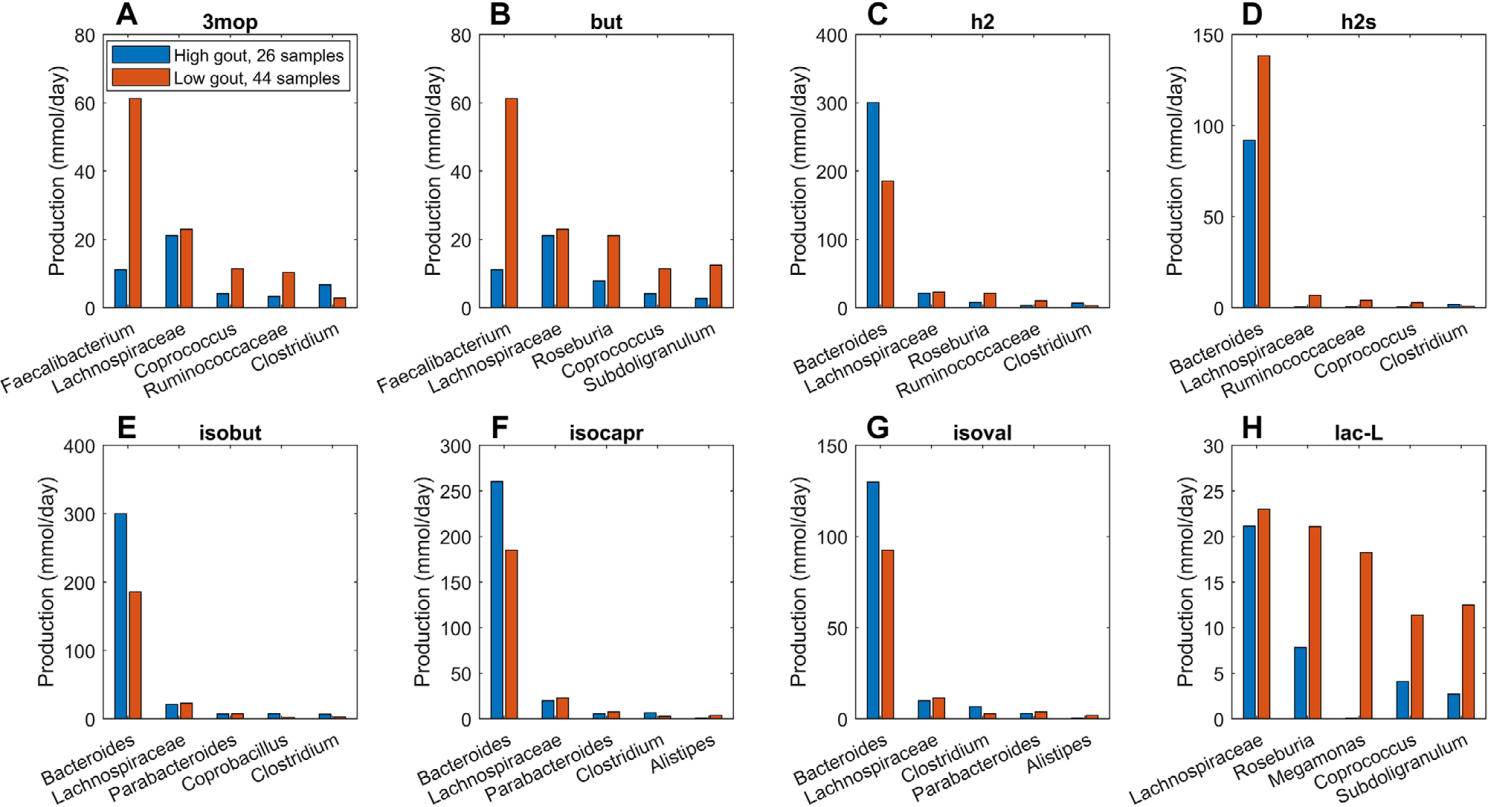
Individual taxa contributions to maximal synthesis of fermentation byproducts differentially produced between the high and low gout clusters from an average EU diet. The byproducts shown from top left to bottom right are 3‐methyl‐2‐oxovaleric acid, butyrate, hydrogen, hydrogen sulfide, isobutyrate, isocaproate, isovalerate and L‐lactate. For each byproduct, the top five taxa are shown in the order of their total production across the two clusters

Similar analyses for differentially produced fermentation byproducts revealed that the potential for significantly elevated synthesis of H_2_, isobutyrate, isocaproate, and isovalerate in the high gout cluster was attributable to *Bacteroides*.(Figure 4) Byproducts not secreted by *Bacteroides* such as 3‐methyl‐2‐oxovaleric acid, butyrate and L‐lactate were predicted to have the potential for significantly elevated production in the low gout cluster due to increased synthesis by more abundant taxa. For example, butyrate was synthesized at higher rates by *Faecalibacterium*, *Roseburia*, *Coprococcus* and *Subdoligranulum* in the low gout cluster. In addition to the recognized importance of microbiota‐derived butyrate for gut health [39, 40, 60] and its previous implication as gout protective [36], these model predictions suggested that butyrate‐producing taxa may contribute to the synthesis of other metabolites possibly involved in gout‐associated microbiota dysbiosis. For example, L‐cysteine was predicted to have the potential for significantly elevated production in the low gout cluster due to enhanced synthesis by *Faecalibacterium* and other butyrate producers (Figure 3). Since H_2_S is a common byproduct of cysteine degradation [41, 42], the ability of butyrate producers to synthesize L‐cysteine could be related to the potential for elevated H_2_S production in the low gout cluster. In addition to H_2_S being a possible inducer of inflammation [43, 44], these predictions suggested a possible role for H_2_S specifically and sulfur‐containing amino acids more generally in the perturbed microbiota of gout patients. Maximal production of the seven non‐differentially produced byproducts was similar between the two clusters with elevated synthesis by *Bacteroides* in the high gout cluster being balanced with increased synthesis by butyrate producers and *Megamonas* in the low gout cluster (Figure S4).

### 3.3 Metabolite crossfeeding supports differential amino acid and fermentation byproduct synthesis

**FIGURE 5:**
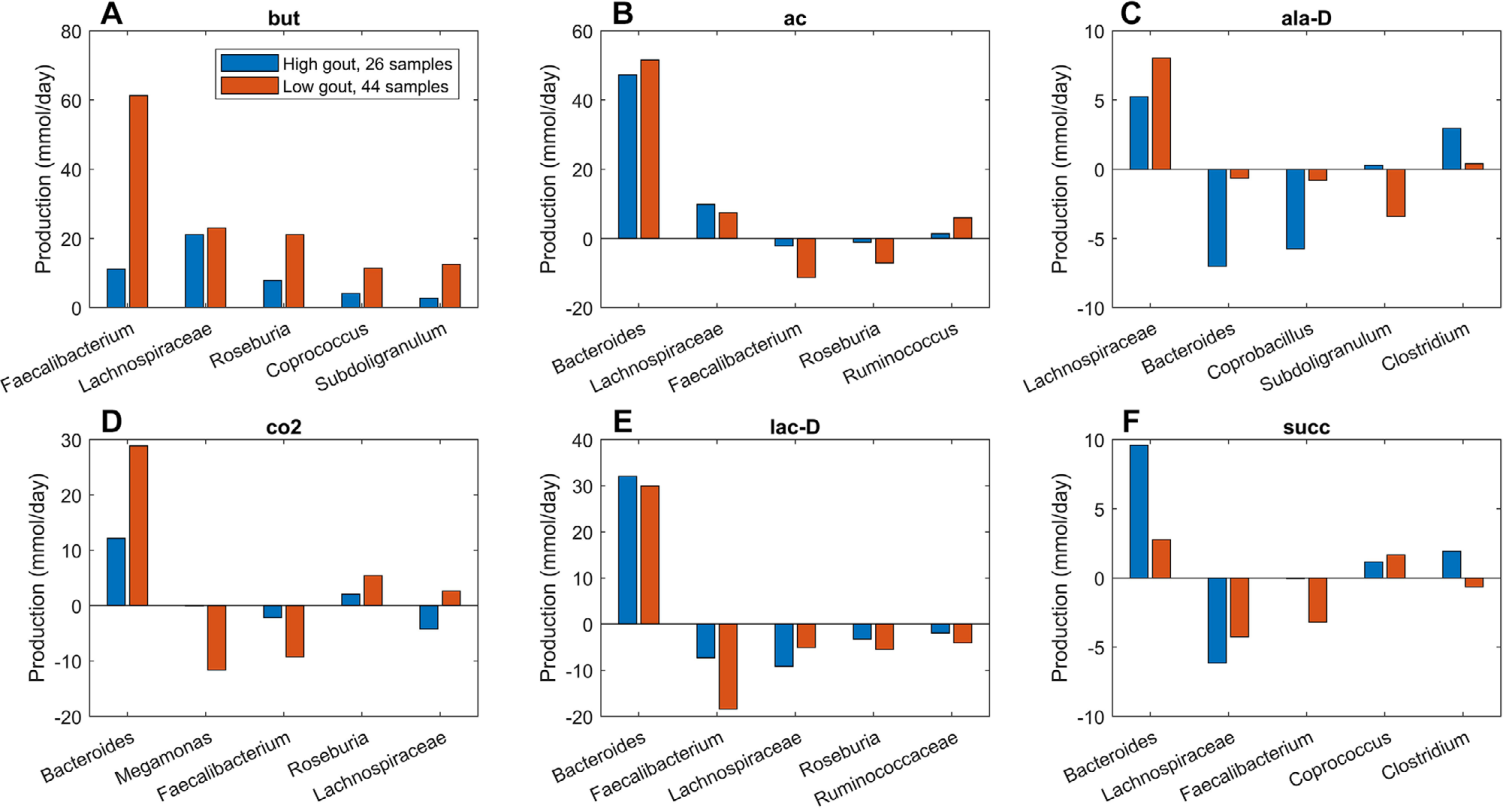
Individual taxa synthesis and uptake of crossfed metabolites for maximal butyrate production from an average EU diet. The metabolites shown from top left to bottom right are butyrate, acetate, D‐alanine, carbon dioxide, D‐lactate and succinate. Each metabolite shown had at least one taxa which satisfied a minimal bound on the metabolite secretion rate and the metabolite uptake rate. For each metabolite, the top five taxa were ordered by the sum of the absolute values of their uptake and secretion rates across the two clusters

The previous model‐based analyses identified butyrate and hydrogen sulfide as putative metabolic markers of gout disease. To investigate interactions between taxa that supported maximal production of these two metabolites, crossfeeding relationships were identified by finding metabolites which were both secreted by at least one taxa and uptaken by at least one other taxa above a defined threshold (5 mmol/d to focus on the largest contributors). Consistent with being more abundant in the low gout cluster, the five taxa mainly responsible for butyrate production were predicted to synthesize more butyrate in these sample communities (Figure 4). However, butyrate production was higher than would be expected based on abundance differences between the two clusters. For example, *Faecalibacterium* was 230% more abundant in the low gout cluster (Figure S1) yet synthesized 550% more butyrate. *Faecalibacterium* was predicted to achieve such elevated butyrate production by exploiting the availability of metabolites secreted from other taxa, most notably acetate, CO_2_, D‐lactate and succinate from *Bacteroides* (Figure 5). Similarly, *Roseburia* utilized acetate and D‐lactate from *Bacteroides* and *Subdoligranulum* utilized D‐alanine from *Lachnospiraceae*. Different crossfeeding relationships were predicted to support maximal production of other fermentation byproducts. For example, *Bacteroides* exploited the availability of secreted L‐alanine and formate to achieve elevated D‐lactate synthesis in the high gout cluster (Figure S5). These results demonstrated the inherent metabolic flexibility of gut bacterial communities and suggested that taxa crossfeeding relationships could be highly context dependent.

**FIGURE 6:**
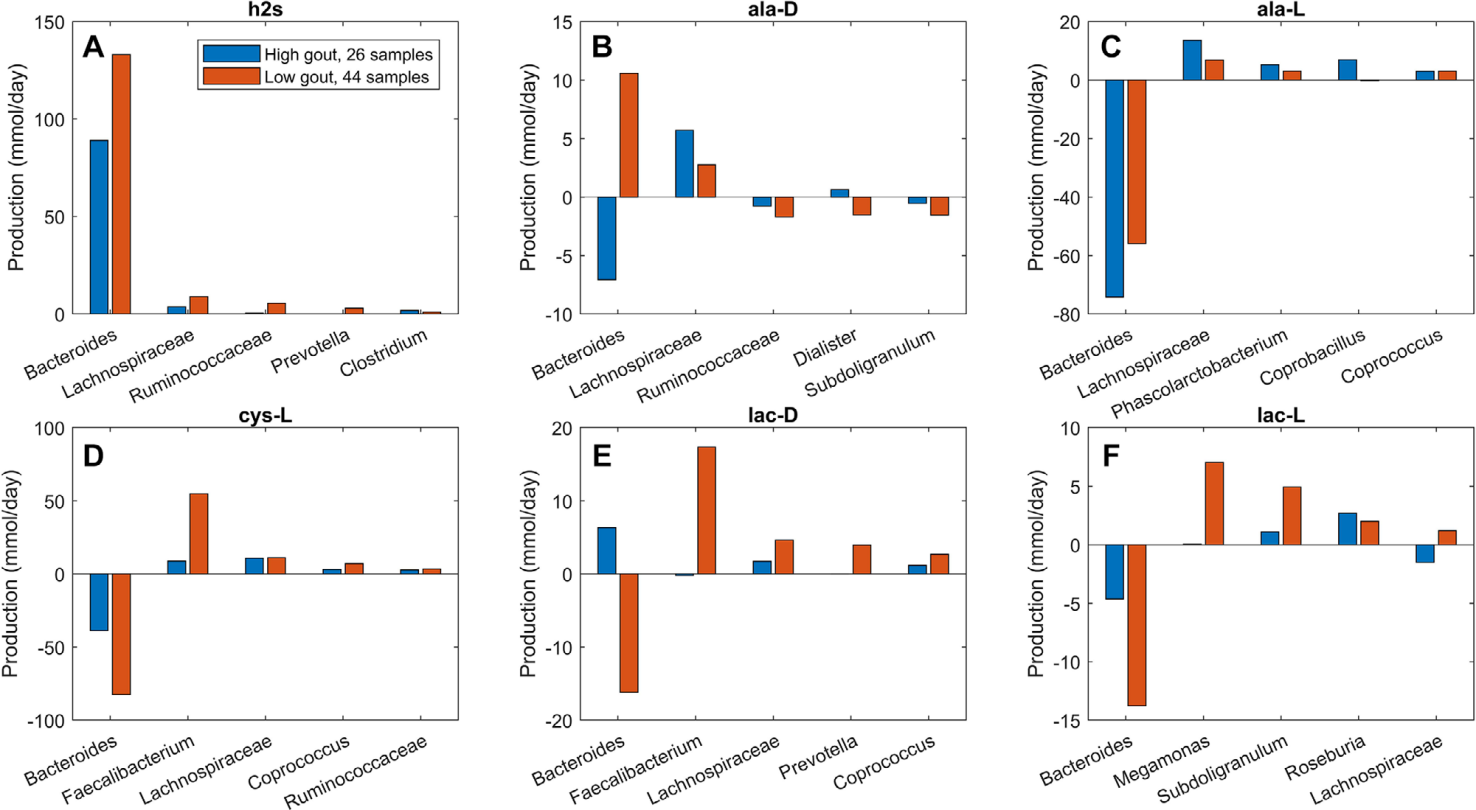
Individual taxa synthesis and uptake of crossfed metabolites for maximal H_2_S production from an average EU diet. The metabolites shown from top left to bottom right are hydrogen sulfide, D‐alanine, L‐alanine, L‐cysteine, D‐lactate and L‐lactate. Each crossfed metabolite shown had at least one taxa which satisfied minimal bounds on the metabolite secretion and uptake rates. For each metabolite, the top five taxa were ordered by the sum of the absolute values of their uptake and secretion rates across the two clusters

Unlike butyrate, H_2_S was predicted to be synthesized almost exclusively by a single taxa, *Bacteroides*. Although *Bacteroides* was 60% more abundant in the high gout cluster than the low gout cluster (Figure 1), the maximal H_2_S production rate was 120% higher in the low gout cluster (Figure 2). Because H_2_S is a product of cysteine degradation [45, 46] and maximal production of L‐cysteine was elevated in the low gout cluster (Figure 2), we hypothesized that L‐cysteine crossfeeding was mainly responsible for differential H_2_S production between the two clusters. When H_2_S production was maximized, L‐cysteine synthesis by *Faecalibacterium* was predicted to be 525% higher in the low gout cluster (Figure 6), which matched the higher *Faecalibacterium* abundance in this cluster (Figure 1). Elevated L‐cysteine synthesis resulted in a 50% increase in H_2_S production by *Bacteroides*, which also preferentially utilized available D‐lactate and L‐lactate in the low gout cluster. Interestingly, D‐alanine crossfeeding supporting maximal H_2_S production was predicted to differ dramatically between the clustered samples with *Bacteroides* consuming the metabolite in the high gout cluster and secreting the metabolite in the low gout cluster. *Faecalibacterium* was predicted to achieve maximal L‐cysteine synthesis through acetate and D‐lactate crossfeeding (Figure S6). Collectively, these results demonstrated that complex relationships may exist between taxa and their metabolic products due to crossfeeding interactions that can be quantified with the type of metabolic modeling approach used in this study.

### 3.4 Different in silico diets generated subtle changes in community metabolism

Previous simulations were performed by constraining community nutrient uptake rates according to an average EU diet (EUD). Diet is known to be strongly associated with gout disease, with high consumption of purine‐rich foods such as meat, poultry and seafoods more likely to result in hyperuricemia and gout development [47, 48, 49]. To investigate the possible effects of dietary nutrients on microbiota metabolism, a high protein diet (HPD) and a high fiber diet (HFD) also were simulated (Table S2). When model‐predicted NMPCs generated with the HPD were clustered, three clusters contained the same samples as when clustering was performed with the EU diet (Figure S7A,B). With a few exceptions (Figure S7C), the two diets generated very similar clustered production of metabolic byproducts (Figure S8).

When partitioned with three clusters, the EUD and HFD generated different sample clustering with the high gout cluster increased from 26 to 31 samples and the low gout cluster decreased from 44 samples to 41 samples (Figure S9A). The number of gouty samples in the two clusters also changed (Figure S9B), but the *Bacteroides*‐dominated cluster remained disproportionally gouty. A rank sum test performed to find metabolites with the potential to be differentially produced between the high gout clusters or the low gout clusters of the two diets identified 10 metabolites, including three metabolites associated with plant polysaccharide degradation (D‐galactose, D‐glucose, D‐maltose) (Figure S9C). Interestingly, H_2_S was no longer differentially produced (Figure S10) even though the HFD contained 84% more L‐cysteine than the EUD.

To further explore how dietary nutrients affected maximal H_2_S production, crossfeeding relationships were identified for the three high gout clusters and for the three low gout clusters generated from the different diets. When the low gout clusters were compared, the HPD was predicted to generate the most H_2_S due to elevated synthesis by *Bacteroides* (Figure S11). By contrast, the HPD generated only slightly higher H_2_S production than the HFD in the high gout clusters (Figure S12). Interestingly, crossfeeding of L‐cysteine from *Faecalibacterium* to *Bacteroides* was elevated for the HFD. These predictions suggested that H_2_S production was partially attributable to reactions associated with sulfur metabolism [50] other than L‐cysteine degradation. The models predicted substantially reduced L‐cysteine crossfeeding and H_2_S production in the high gout clusters compared to the low gout clusters across all three diets, reinforcing cysteine catabolism and H_2_S production as possible markers of gout‐perturbed microbiota.

## 4 DISCUSSION

Recent findings that the gut microbiota are perturbed by gout disease [30, 32–36] motivated this in silico modeling study aimed at identifying putative metabolic features associated with gout development. To this end, bacterial community metabolic models were constructed for 39 gout patients and 39 healthy controls using taxa abundance data generated from stool samples via 16S rRNA gene amplicon sequencing [36]. Model simulations predicted the maximal possible production rates of 409 secreted metabolites for each of the 78 samples. By performing clustering analysis on these model‐predicted metabolic capabilities, the samples were partitioned into a *Bacteroides*‐dominated cluster with a disproportionately large number of gouty samples, a *Faecalibacterium*‐elevated cluster with a disproportionately large number of healthy samples, and a *Prevotella*‐dominated cluster with only six samples. Consistent with the original experimental study [36], these predictions suggested that elevated *Bacteroides* and reduced *Faecalibacterium* abundances were microbiota signatures of gout disease.

To gain mechanistic insights into gut metabolic features associated with gout, secreted metabolites with significantly different maximal synthesis rates in the *Bacteroides*‐dominated, high gout cluster and the *Faecalibacterium*‐elevated, low gout cluster were identified for a simulated EU diet. The low gout cluster was predicted to have elevated synthesis of many metabolites, most notably gut health promoting butyrate and several metabolites associated with sulfur‐containing amino acid metabolism including the L‐cysteine degradation product H_2_S. Detailed analyses of individual taxa contributions to the maximal synthesis of differentially produced amino acids predicted a tradeoff between *Bacteroides* and butyrate producers such as *Faecalibacterium*, *Roseburia*, *Subdoligranulum* and *Coprococcus*. D‐Alanine and L‐alanine elevated in the high gout cluster were synthesized primarily by *Bacteroides*, while amino acids elevated in the low gout cluster were not secreted by *Bacteroides* (L‐isoleucine, L‐tyrosine) or synthesis was more dependent on butyrate producers (L‐cysteine, L‐histidine, L‐methionine). Model predictions associated with the sulfur‐containing amino acids L‐cysteine and L‐methionine were particularly interesting since *Bacteroides* was more abundant in the high gout cluster yet was predicted to have lower maximal synthesis rates of these two sulfur‐containing amino acids.

Similar analyses performed for maximal synthesis of common fermentation byproducts predicted elevated synthesis of isobutyrate, isocarpoate and isovalerate in the high gout cluster resulting from branched‐chain amino acid catabolism. The low gout cluster was predicted to have elevated production of butyrate, L‐lactate and H_2_S, a common end product of L‐cysteine catabolism. The ability of bacterial communities contained in the low gout cluster to generate higher butyrate levels was related to higher abundances of butyrate producers in these samples as well as increased crossfeeding of metabolites such as acetate, D‐alanine, D‐lactate and succinate to the butyrate producers. Butyrate has been widely identified as a gut health promoting metabolite [39, 40], and its reduced production by the gut microbiota has been associated with gout disease in several other studies [31, 36, 51]. Therefore, the computational predictions support the hypothesis that loss of butyrate producers may an important feature of gout‐altered gut microbiota.

Reduced H_2_S production by *Bacteroides* in the high gout cluster was consistent with the prediction of lower total L‐cysteine synthesis in this cluster. By contrast, the low gout cluster was predicted to have elevated H_2_S production due to substantially increased L‐cysteine crossfeeding from *Faecalibacterium* to *Bacteroides*. While *Bacteroides* is not typically viewed as a common genus for H_2_S production [45], several *Bacteroides* strains process the necessary enzymes for cysteine‐to‐H_2_S conversion [57, 58]. H_2_S has been proposed to have both anti‐inflammatory and proinflammatory effects on the human host depending on a number of factors [52, 53, 54, 55], including whether H_2_S is synthesized endogenously by mucosal epithelial cells or derived from the gut microbiota [42, 56]. These in silico predictions revealed an interesting community behavior where synthesis of a potentially inflammatory metabolite (e.g., H_2_S) may be supported by a health‐promoting taxa (e.g., *Faecalibacterium*) crossfeeding a metabolite (e.g., L‐cysteine) to a disease‐promoting taxa (e.g., *Bacteroides*). Collectively, these results suggested that cysteine‐to‐H_2_S conversion by the gut microbiota may represent a novel metabolic signature of gout disease.

Because consumption of high‐purine containing foods such as meat, poultry and fish is a known correlative to gout disease [47, 48, 49], high protein and high fiber diets were simulated and compared to the average EU diet. Unexpectedly, model simulations predicted similar metabolite production profiles for the three in silico diets. These results may have been attributable to limitations of the in silico approach in which sample community compositions were fixed while dietary nutrients were varied, while in reality different diets would be expected to alter microbiota composition. A more consistent analysis could be performed by having 16S‐derived abundance data for both gout patients and healthy controls over a range of known diets.

## CONFLICT OF INTEREST

The author has declared no conflicts of interest.

## Supporting information

Supporting Information

Supporting Information

## Data Availability

The data that supports the findings of this study are available in the supplementary material of this article. The MATLAB codes used to analyze experimental and simulation data are downloadable from the author's research website: www.ecs.umass.edu/che/henson_group/downloads.html
