# Supplementary material for: Interrogation of the perturbed gut microbiota in gouty arthritis patients through in silico metabolic modeling
Source: Eng Life Sci. 2021 Jun 9;21(7):489–501. doi: 10.1002/elsc.202100003 (PMC8257998; doi:10.1002/elsc.202100003)
Supplement: Supplementary file 1 — Supporting Information [file ELSC-21-489-s002.docx]

**Supplemental Figures**

**Figure S1. Analysis of normalized 16S-derived abundance data for samples in which the modeled taxa accounted for at least 90% of the unnormalized abundances.** (A) Taxa which were significantly (FDR < 0.05) more abundant in one sample type versus the other sample type among the 25 most abundant taxa across the 78 samples used. (B) Taxa most highly correlated with the blood uric acid concentration. (C) Taxa with the highest proportionality to *Faecalibacterium*. (D) Principal component plot of the16S-derived abundance data showing gouty and healthy patient samples.

**Figure S2. Differentially produced metabolites between the high and low gout clusters with an average EU diet.** Significant differences in maximal metabolite production rates were determined by applying the Wilcoxon rank sum test (FDR < 0.05) to each metabolite across all samples in the two clusters. In addition to being statistically different, each metabolite shown had an average production rate > 10 mmol/day in at least one cluster and average production rates that differed between the clusters by at least 10%. Metabolite abbreviations are taken from the VMH database ([www.vmh.life](http://www.vmh.life)). Full metabolite names, their associated metabolic pathways and numeric values for their average production rates in each cluster are given in Table S6.

**Figure S3. Individual taxa contributions to maximal synthesis of selected non-differentially produced amino acids in the high and low gout clusters with an average EU diet.** The amino acids shown from top left to bottom right are L-aspartate, L-glutamate, L-leucine, L-phenylalanine, L-tryptophan and L-valine. For each amino acid, the top five taxa are shown in the order of their total production across the two clusters.

**Figure S4. Individual taxa contributions to maximal synthesis of selected non-differentially produced fermentation byproducts in the high and low gout clusters with an average EU diet.** The byproducts shown from top left to bottom right are acetate, carbon dioxide, ethanol, formate, D-lactate, propionate and succinate. For each byproduct, the top five taxa are shown in the order of their total production across the two clusters.

**Figure S5. Individual taxa synthesis and uptake of crossfed metabolites for maximal D-lactate production from an average EU diet.** The metabolites shown from top left to bottom right are D-lactate, acetate, D-alanine, L-alanine, L-cysteine, formate, L-leucine and succinate. Each crossfed metabolite shown had at least one taxa which satisfied minimal bounds on the metabolite secretion and uptake rates. For each metabolite, the top five taxa were ordered by the sum of the absolute values of their uptake and secretion rates across the two clusters.

**Figure S6. Individual taxa synthesis and uptake of crossfed metabolites for maximal L-cysteine production from an average EU diet.** The metabolites shown from top left to bottom right are L-cysteine, acetate, D-alanine, L-alanine, L-glutamate, hydrogen sulfide and L-leucine. Each crossfed metabolite shown had at least one taxa which satisfied minimal bounds on the metabolite secretion and uptake rates. For each metabolite, the top five taxa were ordered by the sum of the absolute values of their uptake and secretion rates across the two clusters.

**Figure S7. Sample clustering and differentially produced metabolites between average EU and high protein diets.** (A) Total samples shared between the three clusters obtained with the average EU diet and the three clusters obtained the high protein diet. (B) Gouty samples shared between the three clusters obtained with the average EU diet and the three clusters obtained the high protein diet. (C) Significant differences in maximal metabolite production rates were determined by applying the Wilcoxon rank sum test (FDR < 0.05) to each metabolite across all samples in the two high gout clusters and the two low gout clusters. In addition to being statistically different, each metabolite shown had an average production rate >10 mmol/day in at least one of the compared clusters and average production rates that differed between the compared clusters by at least 10%. Metabolite abbreviations are taken from the VMH database ([www.vmh.life](http://www.vmh.life)).

**Figure S8. Maximal amino acid and fermentation byproduct synthesis capabilities in the high and low gout clusters from average EU and high protein diets.** (A) Classes of amino acids sharing common metabolic pathways. (B) Common metabolic byproducts of carbohydrate fermentation and amino acid catabolism. Metabolite abbreviations are taken from the VMH database ([www.vmh.life](http://www.vmh.life)).

**Figure S9. Sample clustering and differentially produced metabolites between average EU and high fiber diets.** (A) Total samples shared between the three clusters obtained with the average EU diet and the three clusters obtained the high fiber diet. (B) Gouty samples shared between the three clusters obtained with the average EU diet and the three clusters obtained the high fiber diet. (C) Significant differences in maximal metabolite production rates were determined by applying the Wilcoxon rank sum test (FDR < 0.05) to each metabolite across all samples in the two high gout clusters and the two low gout clusters. In addition to being statistically different, each metabolite shown had an average production rate >10 mmol/day in at least one of the compared clusters and average maximal production rates that differed between the compared clusters by at least 10%. Metabolite abbreviations are taken from the VMH database ([www.vmh.life](http://www.vmh.life)).

**Figure S10. Maximal amino acid and fermentation byproduct synthesis capabilities in the high and low gout clusters from average EU and high fiber diets.** (A) Classes of amino acids sharing common metabolic pathways. (B) Common metabolic byproducts of carbohydrate fermentation and amino acid catabolism. Metabolite abbreviations are taken from the VMH database ([www.vmh.life](http://www.vmh.life)).

**Figure S11. Individual taxa synthesis and uptake of crossfed metabolites for maximal H_2_S production from low gout clusters generated from average European, high protein and high fiber diets.** The metabolites shown from top left to bottom right are hydrogen sulfide, L-alanine, L-cysteine, D-lactate, L-lactate and succinate. Each crossfed metabolite shown had at least one taxa which satisfied minimal bounds on the metabolite secretion and uptake rates for at least one diet. For each metabolite, the top five taxa are ordered by the sum of the absolute values of their uptake and secretion rates across the three diets.

**Figure S12. Individual taxa synthesis and uptake of crossfed metabolites for maximal H_2_S production in high gout clusters generated from average European, high protein and high fiber diets.** The metabolites shown from top left to bottom right are hydrogen sulfide, D-alanine, L-alanine, L-cysteine, D-lactate and succinate. Each crossfed metabolite shown had at least one taxa which satisfied minimal bounds on the maximal metabolite secretion and uptake rates for at least one diet. For each metabolite, the top five taxa are ordered by the sum of the absolute values of their uptake and secretion rates across the three diets.
